# Post-traumatic wrist atrhritis: PRC versus 4-corner fusion

**DOI:** 10.1186/1753-6561-9-S3-A39

**Published:** 2015-05-19

**Authors:** Daniel Herren

**Affiliations:** 1 Department of Hand Surgery, Schulthess Klinik, Zurich, 8008. Switzerland

## Introduction

Painful destruction of the wrist as a consequence of scaphoid non-union advanced collapse (SNAC) or chronic advanced scapho-lunate dissociation (SLAC) is a challenge to treat. In the late stages of either condition, only salvage procedures can be discussed.

The option of proximal row carpectomy (PRC) only remains available in stage II, when the mid-carpal joint is still preserved and the capitate head has an intact cartilage. Four-corner fusion (4CF) is still possible in stages III of these conditions. While PRC is technically less demanding and does not involve the possibility of non-union, different postoperative problems have been described with this technique. This includes radial styloid impingement and pisiform bone impingement syndromes, leading to further interventions.

In addition progressive osteoarthritis at the lunate fossa has been observed, compromising long-term results. In contrast, 4-corner fusion is a technically demanding procedure with a risk of non-union, despite the introduction of new implants. Furthermore malunion with incomplete lunate repositioning may compromise the clinical results.

This study attempts to analyze the results of PRC compared to 4-corner fusion in stages II/III wrists suffering from SNAC or SLAC situations.

## Material and methods

In a retrospective case series, a total of 92 patients with SNAC/SLAC wrist stages II and III were treated with either PRC or 4CF. Sixty-seven wrists, 22 with PRC and 39 with 4-corner fusion, were radiologically and clinically examined after an average follow-up of 5.5 years for PRC and 4.1 years for 4CF. In the 4CF group, 22 patients had a spider plate (Integra™) and 20 patients, a flower plate (KLS Martin™). In none of these cases were additional bone graft needed. In 6 patients with PRC a radial styloidectomy was performed at the primary intervention.

## Results

Neither flexion/extension nor radial/ulnar deviation in the two intervention groups differed significantly postoperative with 55.4°/28.2° in the 4CF group compared to 61°/31.4° in the PRC patients. There was a significant difference (p=0.033) between the wrists with spider plates (49.8°) compared to the wrist with flower plates (61.5°). Grip strength was equal in both groups. Neither pain at rest (0.2 in 4CF vs 0.6 in PRC) nor pain in daily activities (2.0 in 4CF vs 1.9 in PRC) were different in both groups and both measurements was generally low associated with high patients satisfaction. Additionally, the quickDASH (p = 0.042) and the PRWE (p= 0.026) showed a significant difference in favor of the 4-corner fusion. In the group of the PRC, five (20%) impingement cases were observed with the radial styloid and four times with the pisiform. However in the 4CF, 13 wrists (31%) suffered from a dorsal impingement, of which 11 were those treated with a spider plate. A total of 4 revisions were performed in the 4CF group, including one total wrist fusion because of osteoarthritis and two re-fusions due to non-union. In the PRC patients, two cases required removal of the pisiform.

## Conclusion

Over all there was a high patient satisfaction in this difficult to treat wrist situations with both techniques. No significant differences were noted concerning pain reduction, range of motion and grip strength between the two techniques. However the patients with 4-corner fusion had a significantly better subjective outcome. Postoperative problems were closely related to the different implants used. Newer implant designs with low profiles and the option of locking screws are preferable. In addition correct lunate reposition and implant placing is needed to achieve good clinical results and to avoid dorsal impingement.

